# Perilla Oil Supplementation Ameliorates High-Fat/High-Cholesterol Diet Induced Nonalcoholic Fatty Liver Disease in Rats via Enhanced Fecal Cholesterol and Bile Acid Excretion

**DOI:** 10.1155/2016/2384561

**Published:** 2016-08-24

**Authors:** Ting Chen, Fahu Yuan, Hualin Wang, Yu Tian, Lei He, Yang Shao, Na Li, Zhiguo Liu

**Affiliations:** ^1^School of Biology and Pharmaceutical Engineering, Wuhan Polytechnic University, Wuhan, Hubei 430023, China; ^2^School of Medicine, Jianghan University, Wuhan, Hubei, China; ^3^Department of Blood Transfusion, Tongji Hospital, Tongji Medical College, Huazhong University of Science and Technology, Wuhan, Hubei, China

## Abstract

Recent experimental studies and clinical trials have shown that hepatic cholesterol metabolic disorders are closely related to the development of nonalcoholic fatty liver disease (NAFLD). The main goal of this study was to investigate the efficacy of the perilla oil rich in alpha-linolenic acid (ALA) against NASH and gain a deep insight into its potential mechanisms. Rats were fed a high-fat/high-cholesterol diet (HFD) supplement with perilla oil (POH) for 16 weeks. Routine blood biochemical tests and histological staining illustrated that the perilla oil administration improved HFD-induced hyperlipidemia, reduced hepatic steatosis, and inhibited hepatic inflammatory infiltration and fibrosis. Perilla oil also increased fecal bile acid and cholesterol excretion. Hepatic RNA-Seq analysis found that the long time perilla oil supplement notably modified the gene expression involved in cholesterol metabolism. Our results implicate that, after long-term high level dietary cholesterol feeding, rat liver endogenous synthesis of cholesterol and cholesterol-rich low density lipoprotein uptake was significantly inhibited, and perilla oil did not modulate expression of genes responsible for cholesterol synthesis but did increase cholesterol removed from hepatocytes by conversion to bile acids and increased fecal cholesterol excretion.

## 1. Introduction

Nonalcoholic fatty liver disease (NAFLD) is an acquired metabolic stress-induced liver injury, except alcohol and other established impairing liver factors, caused by excessive deposition of fat in the hepatocytes as the main feature of the clinical syndrome, and closely related to genetic susceptibility to insulin resistance, its histological spectrum encompasses simple steatosis (SS); steatosis with necroinflammation, which known as nonalcoholic steatohepatitis (NASH); and cirrhosis [1–5]. As the globalization trends of obesity and related metabolic syndrome, NAFLD has been an important cause of chronic liver disease in developed countries and affluent areas of developing country. Simple steatosis is usually a benign and reversible condition; while the NASH and cirrhosis are much more harmful and may lead to carcinogenesis, blocking the process from steatosis to NASH is crucial for the treatment of NAFLD [6].

The excessive intake of high caloric Western-style diet rich in fat, sugar, and cholesterol and sedentary lifestyle are risk factors for hepatic steatosis; however, the internal mechanism for hepatic steatosis progress to NASH remains unclear. More and more evidences indicated that hepatic free cholesterol accumulation and cholesterol homeostasis play an important role in the development of NASH [7–12]. Hepatic cholesterol metabolism disorder widely exists in NAFLD, resulting in elevated levels of cholesterol in the liver [13, 14]. Multidimensional factors contributing to the dysregulation include increased hydrolysis of cholesteryl esters to free cholesterol [14]; increased endogenous synthesis of cholesterol [15]; increased uptake of cholesterol-rich lipoproteins [14]; decreased conversion of cholesterol to bile acids; and decreased cholesterol excretion [13]. Due to the rigid structure of cholesterol, excess cholesterol affects membrane fluidity and damages membrane proteins function [16]. In addition, the accumulation of excessive cholesterol activates Kupffer cells and hepatic stellate cells, exacerbating liver inflammation, increasing extracellular matrix synthesis, and eventually accelerating the progress of SS to NASH [17–19].

Clinical studies have found that, in NAFLD patients, the serum levels of n-3 polyunsaturated fatty acids (PUFAs), particularly eicosapentaenoic acid (EPA) and docosahexaenoic acid (DHA), were significantly declined [20, 21]. In recent years, the supplement of n-3 PUFAs, such as fish oil rich in EPA and DHA, has been recommended for the prevention and treatment of NAFLD; its inner mechanisms included increasing hepatocellular fatty acid oxidation by activating the peroxisome proliferator activated receptor *α* (PPAR-*α*), inhibiting* de novo* fatty acid synthesis via regulating the expression and maturity of sterol regulatory element-binding protein-1c (SREBP-1c) [22]. However, fish oil could not be used in dietary cooking, which limits its daily application. Several vegetable oils rich in alpha-linolenic acid (ALA) could be another source of dietary n-3 PUFAs. ALA is the most common and accessible n-3 PUFA in the diet; mammals use ALA as a precursor for EPA and DHA [23]. However, the roles of ALA in the modulation of NAFLD are unclear. Although ALA may improve NAFLD via formation functional EPA and DHA, it is also possible that ALA directly actively select receptors, such as PPAR-*α*, influence cellular metabolic processes, or modulate inflammatory pathways.

*Perilla frutescens* is an edible and medicinal plant widely distributed in East Asia and India, and its seeds oil is one of the richest sources of ALA (~60%). The aim of this study was to investigate the effects of ALA rich perilla oil on hepatic steatosis in a high-fat/high-cholesterol diet induced NAFLD rat model and explore its inner mechanism.

## 2. Materials and Methods

### 2.1. Animal Care, Diets and Treatments

The animal experiment follows the procedure presented in our previous study [24]. All experimental procedures were approved by Laboratory Animal Ethics Committee of Wuhan Polytechnic University (ID number: 20141006027). Male 8-9 weeks old Sprague-Dawley rats were randomly assigned to three groups (10 animals per group) as follows: CON: feeding standard chow with 10 kcal% fat; HFD: feeding a Western-style diet with 45 kcal% lard and 2% cholesterol (w/w); POH: feeding a perilla-oil-rich Western-style diet with a total 45 kcal% fat containing 5.5% perilla oil (w/w) and 2% cholesterol (w/w). Perilla oil was obtained from Jilin Shengji Industrial Co. (Jilin, China) which was extracted under a cold-pressed method at 30–48°C. The animals were fed for 16 weeks and then sacrificed. The blood and liver were collected for further analysis.

### 2.2. Serum Biochemical Analyses

Serum total cholesterol (Tch), triglyceride (TG), low-density lipoprotein cholesterol (LDL-C), and high-density lipoprotein cholesterol (HDL-C) levels were measured enzymatically using colorimetric based kits from BioSino Bio-Technology & Science Inc. (Beijing, China), and serum aspartate aminotransferase (AST: (SGOT)) and alanine aminotransferase (ALT: (SGPT)) were tested enzymatically using kits from Nanjing Jiancheng Institute of Biological Engineering (Nanjing, China). All tests were performed in triplicate according to the standard process by spectrophotometry.

### 2.3. Fecal Lipids and Bile Acid Analysis

Fecal bile acid analysis referenced Modica and colleagues reported method [25]; briefly, collected feces was crushed after vacuum freeze drying; the feces powder was mixed with 2 mg/mL sodium tetrahydridoborate in ethanol and shaking for half an hour. Samples were filtered with 0.45 *μ*m membrane filter and dried under nitrogen and then were redissolved in methanol and measured by the Total Bile Acids Assay kit from Nanjing Jiancheng Institute of Biological Engineering (Nanjing, China). Total fecal lipids were extracted by Soxhlet extractor with ether and n-hexane as solvents, assessed via gravimetric analysis. To assay fecal cholesterol, a method described by Modica et al. was used to extract cholesterol from feces [25], and cholesterol level was measured using a colorimetric based kits from Nanjing Jiancheng Institute of Biological Engineering (Nanjing, China).

### 2.4. Histological Studies

Take the same parts of the rat liver (left lobe), fixed with 4% paraformaldehyde and serial sectioned at 5 *μ*m thickness, stained with hematoxylin and eosin or Masson's trichrome. NAFLD activity score was assessed by a pathologist who was blinded to the study design, according to the scoring system proposed by Kleiner et al. [26].

### 2.5. mRNA Library Construction, Sequencing, and Data Analysis

As previously described [24], total RNA extracted using Trizol reagent from rat livers, the pooled sample for each group was prepared using an equal amount of total RNA from each individual [27, 28]. High-throughput sequencing was performed on Illumina Hiseq 2000 system. The difference of expression greater than twofold gene lists were submitted to DAVID [29] web server (https://david.abcc.ncifcrf.gov/) for enrichment analysis.

### 2.6. Quantitative RT-PCR

The RNA-seq data were verified by quantitative real-time PCR (qPCR); we selected high, intermediate, or low expressing transcripts for analysis as Merrick et al. described [30]. RT-qPCR analysis was performed on an ABI StepOnePlus*™* (Applied Biosystems, CA, USA), difference of gene expression using the 2^−ΔΔCt^ method by normalizing to* GAPDH* expression which did not vary significantly with high-fat diet treatment.

### 2.7. Western Blot Analysis

For ~5 mg piece of rat liver tissue, we added ~300 *μ*L of ice cold radioimmunoprecipitation assay (RIPA) buffer rapidly to the tube, homogenized with an electric homogenizer, and then centrifuged for 30 min at 10,000 rpm at 4°C to collect the protein sample. Equal amounts of protein were loaded and separated on gels and electrotransferred to PVDF membranes. Antibodies of SREBP2, FXR, LXR, VDR, and GAPDH were obtained from Santa Cruz Biotechnology, Inc. (Cambridge, MA, USA). Densitometry was determined using Image J v 1.30 obtained from NIH (Bethesda, MD, USA).

## 3. Results

### 3.1. Perilla Oil Ameliorated HFD-Induced Hyperlipidemia

**Figure 1:**
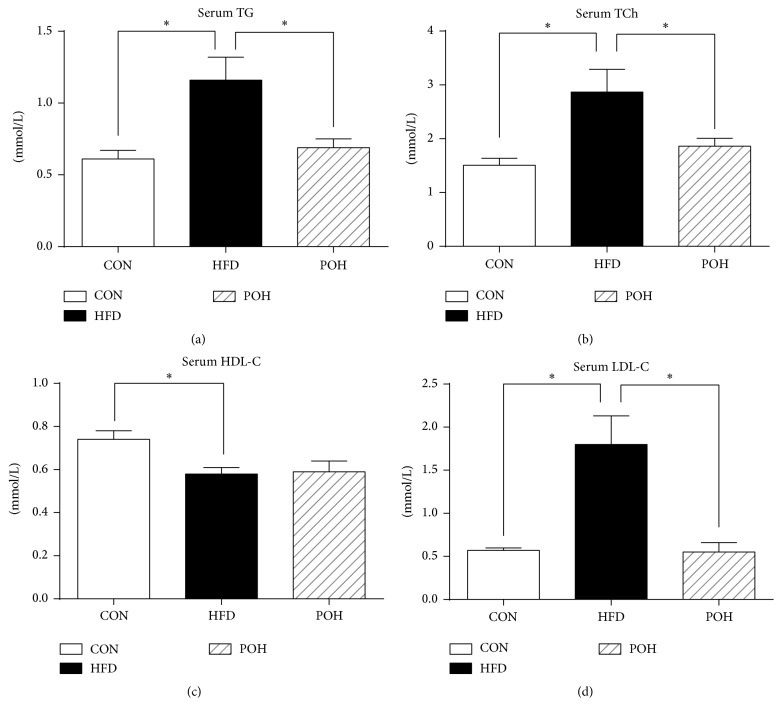
Serum biochemical analysis. Serum was analyzed for (a) triglycerides (TG), (b) total cholesterol (TCh), (c) high-density lipoprotein cholesterol (HDL-C), and (d) low-density lipoprotein cholesterol (LDL-C), ^*∗*^ *P* < 0.05.

The serum lipids assay showed tha,t compared with CON group, long-term HFD-fed animals had significantly higher grade of the serum TG, TCh, and LDL-C as we have shown in previous study [24]. The effects of HFD on serum lipids were abolished by perilla oil supplement; compared with HFD group, the serum TG, TCh, and LDL-C concentration in POH group were decreased by 43%, 32%, and 28%, respectively (*P* < 0.05) (Figure 1). In general, 16 weeks' HFD feeding induced typical hyperlipidemia, which was rescued by ALA-rich perilla oil.

### 3.2. Perilla Oil Increases Fecal Lipids, Cholesterol, and Bile Acid Excretion

**Figure 2:**
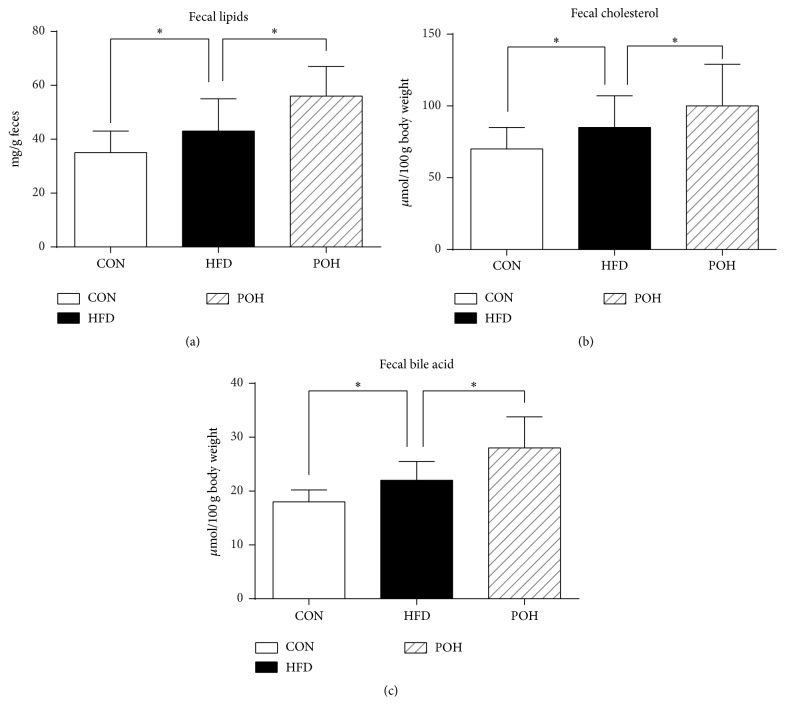
Perilla oil increased fecal lipids, cholesterol, and bile acid excretion. Fecal (a) total lipids, (b) cholesterol, and (c) bile acid were analyzed. ^*∗*^ *P* < 0.05.

High-fat and high-cholesterol feeding for 16 weeks significantly increase rat fecal lipids, cholesterol, and bile acid levels compared to the rats fed the control diet, while perilla oil further enhanced their excretion (Figure 2).

### 3.3. Perilla Oil Alleviated Liver Injury in Rats Fed HFD

**Figure 3:**
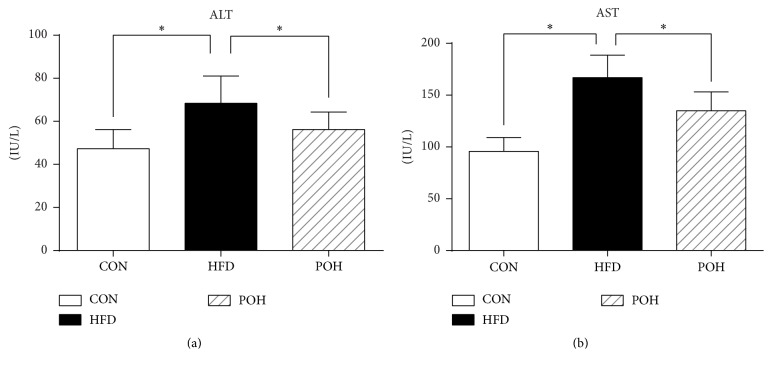
Perilla oil decreased serum ALT and AST levels. Serum alanine aminotransferase (ALT: (SGPT)) and aspartate aminotransferase (AST: (SGOT)) were analyzed. ^*∗*^ *P* < 0.05.

**Figure 4:**
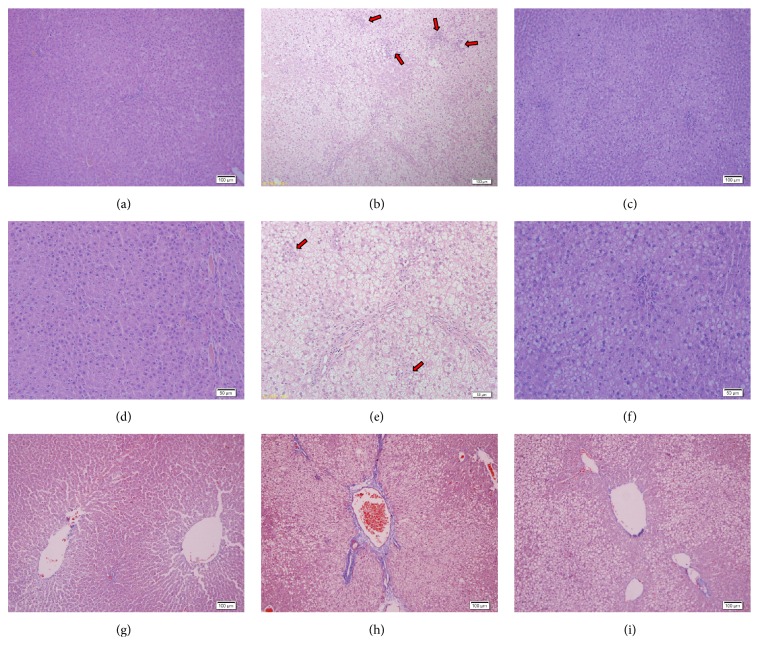
Effect of perilla oil and high-fat diet on rat liver histology. The representative photographs are as follows: (a)–(c) H&E staining photomicrographs of CON, HFD, and POH liver section (×100); (d)–(f) H&E staining photomicrographs of CON, HFD, and POH liver section (×200); (g)–(i) Masson's Trichrome staining photomicrographs of CON, HFD, and POH liver section (×100). The red arrow indicates the inflammatory infiltration area and blue parts as collagen in Masson's Trichrome staining.

**Figure 5:**
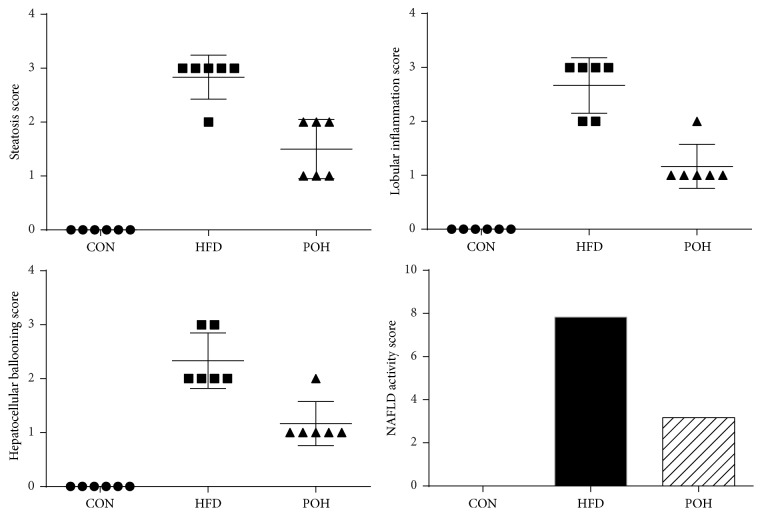
NAFLD activity score (NAS). NAFLD activity scores (NAS): the unweighted sum of steatosis, lobular inflammation, and hepatocellular ballooning, NAS of >5 correlated with a diagnosis of NASH.

Elevated serum aminotransferase levels typically prompts liver injury. The ALT and AST levels in rats fed HFD were significantly increased compared with CON, whereas the activity of ALT and AST in POH group was decreased by 18% and 19%, respectively, compared with HFD (Figure 3). H&E staining illustrated that POH dramatically reduced liver steatosis compared with that of HFD group. Furthermore, POH largely diminished liver inflammation infiltration around portal area (Figures 4(a)–4(f)). Masson's staining showed POH-rescued HFD-induced hepatic fibrosis (Figures 4(g)–4(i)). Hepatic histological NAFLD activity score indicated that 16-week HFD feeding induced NASH in rats (NAS = 7.83), but the rats in the POH group only developed simple steatosis without steatohepatitis (NAS = 3.33) (Figure 5).

### 3.4. Summary and Validation of RNA-seq

We used paired-end RNA-seq approach which generated about 263 million reads in three groups of rat liver and harvested ~3 gigabases (Gb) of sequence per group. More than 60% reads can be aligned to the UCSC rat reference genome (http://hgdownload.cse.ucsc.edu/downloads.html#rat) (Table 1). We set single transcript FPKM value >0.05 and expressed it in at least one of the three groups termed as expressed transcript. Thus, we identified 17,939, 18,098, and 17,664 transcripts in CON, HFD, and POH, respectively (Table 1).

**Figure 6:**
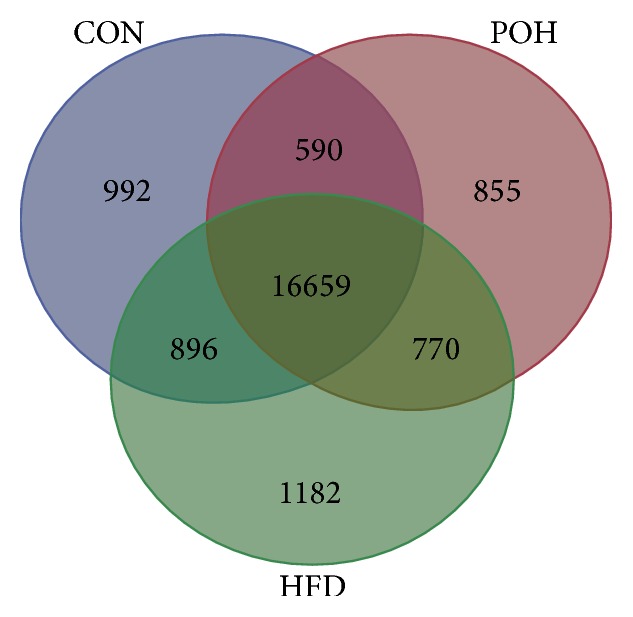
Comparison of the transcriptomes of three group liver tissues.

**Figure 7:**
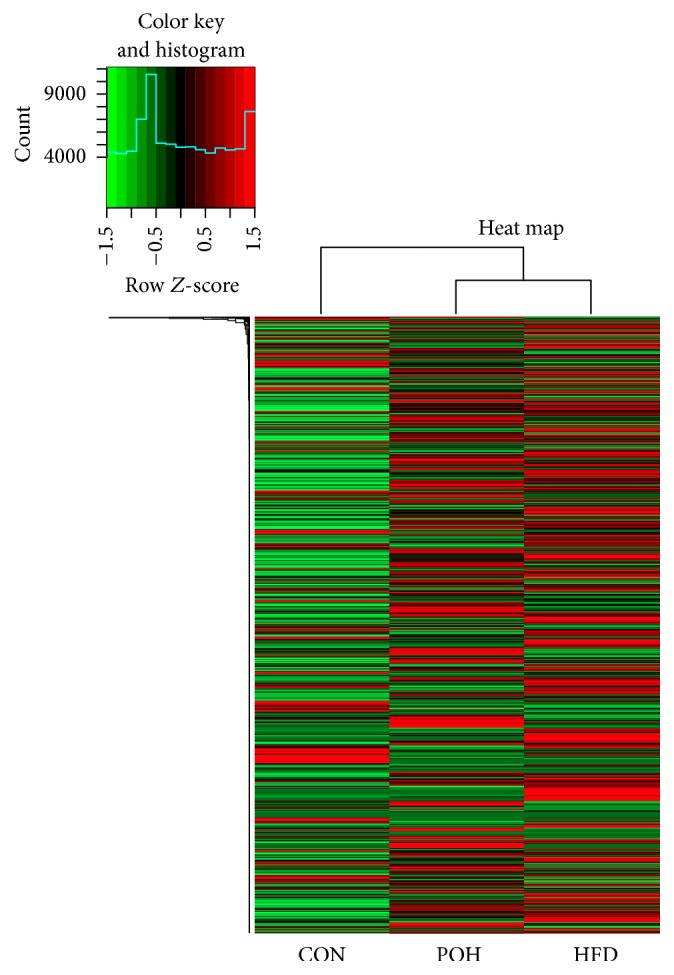
Hierarchical clustering using all expressed transcripts.

A total of 16,659 transcripts were coexpressed in the three groups; there are 992, 855, and 1182 transcripts expression separate in CON, POH, and HFD, respectively (Figure 6). Clustering analysis of the three groups showed that POH and HFD are closer to each other than to CON, which also highlights the different transcriptome features of POH compared with HFD (Figure 7).

**Figure 8:**
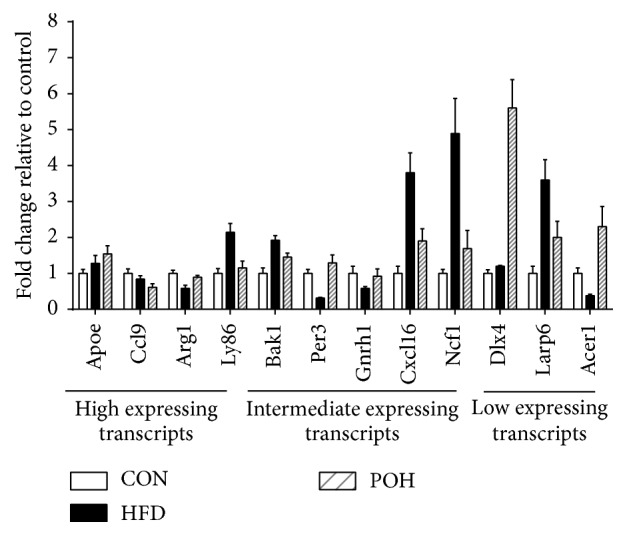
The qPCR results of selected genes. The mRNA expression levels were examined by qPCR. Values are expressed as mean ± SD; *n* = 6 in each treatment group.

The expression of high, intermediate, or low expressing transcripts was examined by qPCR (Figure 8). The results of qPCR were consistent with our RNA-seq data.

### 3.5. Functional Enrichment of DEGs in POH Compared with That of HFD Feeding Rats

**Figure 9:**
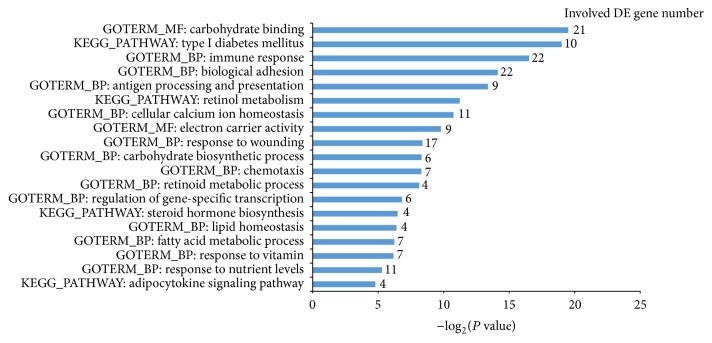
GO (Gene Ontology) and pathway categories enriched for POH versus HFD DE transcripts.

To analyze DEGs, we set fold change >2 and *P* value <0.01 to select DEGs. Compared with HFD group, 678 DEGs were found in POH group; among them, there are 266 genes' expression where increased DAVID clustering analysis result showed that, compared with HFD group, the upregulated DEGs clusters in POH group are mainly involved in electron carrier activity, steroid hormone biosynthesis, fatty acid metabolic process, response to nutrient, and carbohydrate biosynthetic process, while downregulated gene clusters are involved in processes such as biological adhesion, immune response, carbohydrate binding, cellular calcium ion homeostasis, chemotaxis, and response to wounding (Figure 9).

### 3.6. Hepatic Cholesterol and Bile Acid Synthesis and Transport Related mRNA and Protein Expression

**Figure 10:**
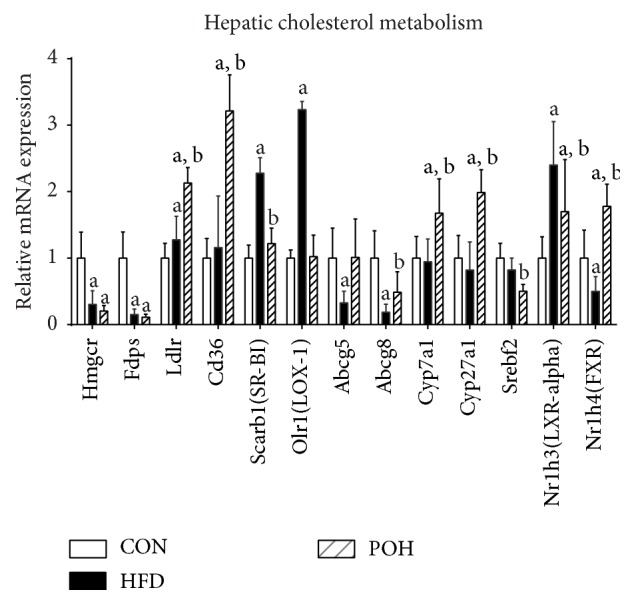
Hepatic expression of genes involved in cholesterol and bile acid synthesis and transport. Values are expressed as mean ± SD; *n* = 10 in each treatment group. a, versus CON group; b, versus HFD group, *P* < 0.05.

As Figure 10 shows,* Hmgcr* mRNA expression in liver in both HFD and POH rat was significantly decreased compared to that in control group (*P* < 0.05); while there was no significantly expression between POH and HFD, the same trend was also observed in* Fadps* and* Srebpf2*. The expressions of* Cyp7a1* and* Cyp27a1* were increased in POH group compared with HFD group (*P* < 0.05), whereas they were not obvious between HFD and CON.* Abcg5/8* mRNA expression in POH was significantly increased compared to that in HFD after 16 weeks (*P* < 0.05), which were decreased in HFD compared with CON. The expressions of* Scarb1* and* Olr1* were significantly increased in HFD compared to that in other groups (*P* < 0.05).

**Figure 11:**
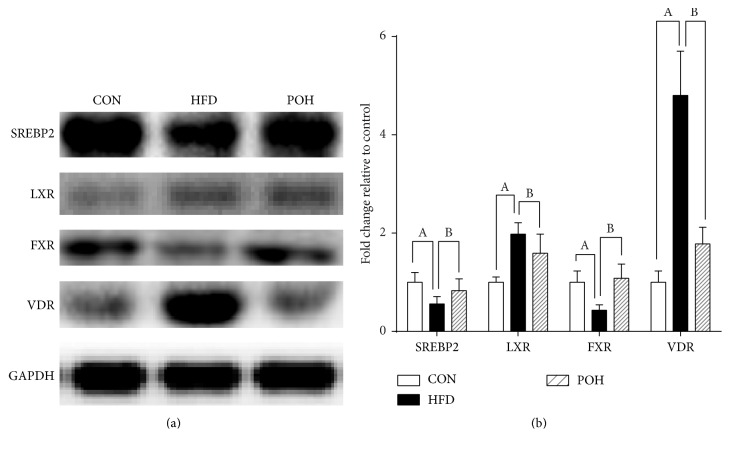
Hepatic transfactors expression involved in cholesterol and bile acid synthesis and transport. (a) Western blot analysis of SREBP2, LXR, FXR, and VDR expression in liver. (b) The SREBP2, LXR, FXR, and VDR expression levels were quantitatively analyzed with Image J. Values are expressed as mean ± SD; *n* = 6 in each treatment group. A, versus CON group; B, versus HFD group, *P* < 0.05.

After 16 weeks, the FXR and SREBP2 protein levels in liver of HFD were significantly inhibited compared with that of the control group, and they were reversed in POH (Figure 11, *P* < 0.05). Hepatic LXR levels increased in HFD and it was inhibited in POH. The VDR levels in liver of HFD increased about 5 times compared to CON (*P* < 0.05), and it was reversed to normal levels in the presence of perilla oil.

## 4. Discussion

In the present study, we revealed that the ALA-rich perilla oil feeding slowed down the development of NAFLD caused by high-fat and high-cholesterol diet feeding; the concentrations of blood triglyceride, total cholesterol, and LDL-C in perilla oil group were significantly lower than that in the HFD group and similar as the level of the control group. Histological staining showed that perilla oil supplement reduced the HFD-induced hepatic steatosis, and the degree of inflammation and fibrosis in the POH group was slightly lower than that of the HFD group, suggesting that perilla oil consumption can modestly alleviate the Western-style diet induced lipid accumulation, inflammation, and fibrogenesis in liver. To further explore the molecular mechanisms, in this study, we applied a next-generation high-throughput sequencing technology to investigate the hepatic mRNA expression difference between the rats in HFD and POH group. Our previous work has showed the HFD feeding regulated the hepatic expression of genes involved in lipid metabolism, cholesterol metabolism, and hepatic inflammation [24]. Here, we further demonstrated that perilla oil consumption reversed the effects of HFD on the hepatic gene expressions, particularly in terms of genes associated with hepatic cholesterol metabolism.

In hepatocytes, intracellular cholesterol homeostasis is maintained through a coordinate network involving cholesterol-sensors and nuclear transcription factors regulating cholesterol synthesis, esterification, uptake, intracellular transport, and excretion [13]. In the present study, 16 weeks' HFD (2% cholesterol, w/w) feeding induced hypercholesterolemia and hepatic cholesterol overload in rats, and perilla oil supplement significantly reduced the cholesterol content in blood and liver, especially in the form of low-density lipoprotein cholesterol (LDL-C). In hepatocyte, the steady state of cholesterol content is attributed to the complex regulation proteins such as cholesterol receptors and nuclear factors involved in cholesterol synthesis, esterification, uptake, and secretion. The major sources of hepatic cholesterol are uptake from plasma lipoprotein and* de novo* synthesis. The 3-hydroxy-3-methylglutaryl-coenzyme reductase (HMGCoAR) is the key rate-limiting enzyme in cholesterol synthesis [31]. HFD intake decreased its expression in liver, and the expressions of other enzymes in charge of cholesterol synthesis such as phosphomevalonate kinase and farnesyl diphosphate synthase were also decreased. Several hepatocyte membrane receptors such as FAT/CD36, Ldlr, LOX-1, and SR-B1 are involved in cholesterol enriched lipoproteins uptake. Perilla oil consumption elevated the expression of* Ldlr* and Fat/*Cd36* but reduced the expression of* Olr1* and* Scarb1* in diet induced NAFLD animals (Figure 10). CD36 is a multifunctional receptor that takes part in the uptake of oxLDL, VLDL, I/IV collagen, and FFAs. The overexpression of CD36 as well as Ldlr in hepatocyte is positively related to steatosis in NAFLD patients. In the diet induced NAFLD model, the expressions of* Cd36* and* Ldlr* had no difference between HFD group and control group, while perilla oil stimulated their expression (Figure 9), which may be due to scavenging of circulating FFAs and LDL-C. ABC protein family is associated with small molecule transport, and in mammalian cells, ABCG5 and ABCG8 are involved in cholesterol excretion [32]. In the study the results showed the HFD downregulated the expression of* Abcg5/8*, which have been reported earlier, while the effects were abrogated in POH group (Figure 10). It suggests dietary perilla oil intake contributes to hepatic cholesterol excretion. Several studies have pointed out perilla oil intake leads to increase of bile acid excretion via upregulating the expression of CYP7A1 and CYP27A1, two key enzymes in charge of bile acid production [31]. In the present study, the expressions of* Cyp7a1* and* Cyp27a1* were reduced in HFD group and increased in POH group (Figure 9); it demonstrates that the cholesterol-lowering effects of perilla oil partly are attributed to the elevated bile acid excretion. Three nuclear transcription factors regulating cholesterol metabolism have been linked to NAFLD: sterol regulatory element-binding protein (SREBP-2), farnesoid X receptor (FXR), and liver X receptor (LXR) [33]. LXR regulates hepatic triglyceride and cholesterol metabolism in rodents; increased expression of hepatic LXR in humans has been reported [34]; our western blotting results showed increased LXR expression in high-fat and high-cholesterol diet rat, which was reversed by perilla oil supplementation. FXR was the master regulator of bile acid synthesis [35]; it suppresses bile acid synthesis by inhibiting CYP7A1 via inducing intestinal epithelial expression of FGF15 [36]; perilla oil increased FXR expression in POH, which was inhibited in HFD.

Recently, the role of vitamin D receptor (VDR) in NAFLD has been investigated; evidence showed that vitamin D deficiency contributes to the development of NAFLD [37]; vitamin D levels are related to the histological severity of steatosis [38, 39]. As Bozic et al. [40] recently published research results, we also observed the elevation of the VDR expression in the HFD; furtherly its expression in perilla oil treatment group significantly reduced; however, the molecular mechanisms underlined require further investigation.

In conclusion, the present study demonstrated that chronic HFD feeding induced hepatic lipid overload, hepatocellular cholesterol metabolic imbalance in the progress of NAFLD. The perilla oil supplement rescued the HFD-induced steatosis and depressed hepatic inflammation; both contributed to the amelioration of diet induced NAFLD progress. The results indicated perilla oil rich in ALA can regulate the expression of multiple nuclear transcription factors, including VDR, thus alleviating liver cholesterol overload and metabolic disorders, and perilla oil can be a potential dietary therapeutic tool against NAFLD.

## Figures and Tables

**Table 1:** Summary of RNA-seq alignment.

Group	COH	POH	HFD
Total sequenced reads	34354375	24749297	28713141
Total aligned reads	31325266	21899853	25396543
Uniquely aligned reads	28340661	20058424	23119703
Multiple aligned reads	2984605	1841429	2276840
Unmapped reads	3029109	2849444	3316598
Expressed transcripts	17939	17664	18098

## References

[1] Verbeek J., Cassiman D., Lannoo M. (2013). Treatment of non-alcoholic fatty liver disease: can we already face the epidemic?. *Acta Gastro-Enterologica Belgica*.

[2] Fan J.-G., Farrell G. C. (2009). Epidemiology of non-alcoholic fatty liver disease in China. *Journal of Hepatology*.

[3] Farrell G. C., Wong V. W.-S., Chitturi S. (2013). NAFLD in Asia—as common and important as in the West. *Nature Reviews Gastroenterology and Hepatology*.

[4] Gariani K., Philippe J., Jornayvaz F. R. (2013). Non-alcoholic fatty liver disease and insulin resistance: from bench to bedside. *Diabetes and Metabolism*.

[5] Masuoka H. C., Chalasani N. (2013). Nonalcoholic fatty liver disease: an emerging threat to obese and diabetic individuals. *Annals of the New York Academy of Sciences*.

[6] McCullough A. J. (2006). Pathophysiology of nonalcoholic steatohepatitis. *Journal of Clinical Gastroenterology*.

[7] Te Sligte K., Bourass I., Sels J. P., Driessen A., Stockbrugger R. W., Koek G. H. (2004). Non-alcoholic steatohepatitis: review of a growing medical problem. *European Journal of Internal Medicine*.

[8] Musso G., Gambino R., Pacini G., De Michieli F., Cassader M. (2009). Prolonged saturated fat-induced, glucose-dependent insulinotropic polypeptide elevation is associated with adipokine imbalance and liver injury in nonalcoholic steatohepatitis: dysregulated enteroadipocyte axis as a novel feature of fatty liver. *American Journal of Clinical Nutrition*.

[9] Tomita K., Teratani T., Yokoyama H. (2011). Plasma free myristic acid proportion is a predictor of nonalcoholic steatohepatitis. *Digestive Diseases and Sciences*.

[10] Lomonaco R., Ortiz-Lopez C., Orsak B. (2011). Role of ethnicity in overweight and obese patients with nonalcoholic steatohepatitis. *Hepatology*.

[11] Musso G., Gambino R., De Michieli F. (2003). Dietary habits and their relations to insulin resistance and postprandial lipemia in nonalcoholic steatohepatitis. *Hepatology*.

[12] Gentile C. L., Pagliassotti M. J. (2008). The role of fatty acids in the development and progression of nonalcoholic fatty liver disease. *Journal of Nutritional Biochemistry*.

[13] Musso G., Gambino R., Cassader M. (2013). Cholesterol metabolism and the pathogenesis of non-alcoholic steatohepatitis. *Progress in Lipid Research*.

[14] Min H.-K., Kapoor A., Fuchs M. (2012). Increased hepatic synthesis and dysregulation of cholesterol metabolism is associated with the severity of nonalcoholic fatty liver disease. *Cell Metabolism*.

[15] Simonen P., Kotronen A., Hallikainen M. (2011). Cholesterol synthesis is increased and absorption decreased in non-alcoholic fatty liver disease independent of obesity. *Journal of Hepatology*.

[16] Ioannou G. N. (2016). The role of cholesterol in the pathogenesis of NASH. *Trends in Endocrinology and Metabolism*.

[17] Bieghs V., Walenbergh S. M. A., Hendrikx T. (2013). Trapping of oxidized LDL in lysosomes of Kupffer cells is a trigger for hepatic inflammation. *Liver International*.

[18] Bieghs V., Hendrikx T., Van Gorp P. J. (2013). The cholesterol derivative 27-hydroxycholesterol reduces steatohepatitis in mice. *Gastroenterology*.

[19] Tomita K., Teratani T., Suzuki T. (2014). Acyl-CoA: cholesterol acyltransferase 1 mediates liver fibrosis by regulating free cholesterol accumulation in hepatic stellate cells. *Journal of Hepatology*.

[20] Araya J., Rodrigo R., Videla L. A. (2004). Increase in long-chain polyunsaturated fatty acid n-6/n-3 ratio in relation to hepatic steatosis in patients with non-alcoholic fatty liver disease. *Clinical Science*.

[21] Allard J. P., Aghdassi E., Mohammed S. (2008). Nutritional assessment and hepatic fatty acid composition in non-alcoholic fatty liver disease (NAFLD): a cross-sectional study. *Journal of Hepatology*.

[22] El-Badry A. M., Graf R., Clavien P.-A. (2007). Omega 3—Omega 6: what is right for the liver?. *Journal of Hepatology*.

[23] Brenna J. T. (2002). Efficiency of conversion of alpha-linolenic acid to long chain n-3 fatty acids in man. *Current Opinion in Clinical Nutrition and Metabolic Care*.

[24] Yuan F., Wang H., Tian Y. (2016). Fish oil alleviated high-fat diet-induced non-alcoholic fatty liver disease via regulating hepatic lipids metabolism and metaflammation: a transcriptomic study. *Lipids in Health and Disease*.

[25] Modica S., Murzilli S., Moschetta A. (2011). Characterizing bile acid and lipid metabolism in the liver and gastrointestinal tract of mice. *Current Protocols in Mouse Biology*.

[26] Kleiner D. E., Brunt E. M., Van Natta M. (2005). Design and validation of a histological scoring system for nonalcoholic fatty liver disease. *Hepatology*.

[27] Long Q., Jeffares D. C., Zhang Q. (2011). PoolHap: inferring haplotype frequencies from pooled samples by next generation sequencing. *PLoS ONE*.

[28] Konczal M., Koteja P., Stuglik M. T., Radwan J., Babik W. (2014). Accuracy of allele frequency estimation using pooled RNA-Seq. *Molecular Ecology Resources*.

[29] Huang D. W., Sherman B. T., Lempicki R. A. (2009). Systematic and integrative analysis of large gene lists using DAVID bioinformatics resources. *Nature Protocols*.

[30] Merrick B. A., Phadke D. P., Auerbach S. S. (2013). RNA-Seq profiling reveals novel hepatic gene expression pattern in aflatoxin B1 treated rats. *PLoS ONE*.

[31] Langston T. B., Hylemon P. B., Grogan W. M. (2005). Over-expression of hepatic neutral cytosolic cholesteryl ester hydrolase in mice increases free cholesterol and reduces expression of HMG-CoAR, CYP27, and CYP7A1. *Lipids*.

[32] Baldán Á., Bojanic D. D., Edwards P. A. (2009). The ABCs of sterol transport. *Journal of Lipid Research*.

[33] Cave M. C., Clair H. B., Hardesty J. E. (2016). Nuclear receptors and nonalcoholic fatty liver disease. *Biochimica et Biophysica Acta (BBA)-Gene Regulatory Mechanisms*.

[34] Ahn S. B., Jang K., Jun D. W., Lee B. H., Shin K. J. (2014). Expression of liver X receptor correlates with intrahepatic inflammation and fibrosis in patients with nonalcoholic fatty liver disease. *Digestive Diseases and Sciences*.

[35] Evans R. M., Mangelsdorf D. J. (2014). Nuclear receptors, RXR, and the big bang. *Cell*.

[36] Inagaki T., Choi M., Moschetta A. (2005). Fibroblast growth factor 15 functions as an enterohepatic signal to regulate bile acid homeostasis. *Cell Metabolism*.

[37] Alvarez J. A., Ashraf A. (2010). Role of vitamin D in insulin secretion and insulin sensitivity for glucose homeostasis. *International Journal of Endocrinology*.

[38] Zúñiga S., Firrincieli D., Housset C., Chignard N. (2011). Vitamin D and the vitamin D receptor in liver pathophysiology. *Clinics and Research in Hepatology and Gastroenterology*.

[39] Kwok R. M., Torres D. M., Harrison S. A. (2013). Vitamin D and nonalcoholic fatty liver disease (NAFLD): is it more than just an association?. *Hepatology*.

[40] Bozic M., Guzmán C., Benet M. (2016). Hepatocyte vitamin D receptor regulates lipid metabolism and mediates experimental diet-induced steatosis. *Journal of Hepatology*.

